# Red cell distribution width associations with clinical outcomes: A population-based cohort study

**DOI:** 10.1371/journal.pone.0212374

**Published:** 2019-03-13

**Authors:** Marcello Tonelli, Natasha Wiebe, Matthew T. James, Christopher Naugler, Braden J. Manns, Scott W. Klarenbach, Brenda R. Hemmelgarn

**Affiliations:** 1 University of Calgary, Calgary, Alberta, Canada; 2 Alberta Kidney Disease Network, Alberta, Canada; 3 University of Alberta, Edmonton, Alberta, Canada; International University of Health and Welfare, School of Medicine, JAPAN

## Abstract

**Importance:**

Higher levels of red cell distribution width (RDW) are associated with adverse outcomes, especially in selected cohorts with or at risk for chronic disease. Whether higher RDW or the related parameter standard deviation of the red blood cell distribution (SD-RBC) can predict a broader range of outcomes in the general population is unknown.

**Objective:**

To evaluate the association of RDW and SD-RBC with the risk of adverse outcomes in people from the general population.

**Design:**

Population-based retrospective cohort study.

**Setting:**

Health care system in a Canadian province (Alberta).

**Participants:**

All 3,156,863 adults living in Alberta, Canada with at least one measure of RDW and SD-RBC between 2003 and 2016. Data were analyzed in September 2018.

**Exposure:**

RDW and SD-RBC, classified into percentiles (<1, 1–5, 5–25, 25–75, 75–95, 95–99, >99).

**Main outcomes:**

All-cause death, first myocardial infarction, first stroke or transient ischemic attack, placement into long-term care (LTC), progression to renal replacement therapy (initiation of chronic dialysis or pre-emptive kidney transplantation), incident solid malignancy, and first hospitalization during follow-up.

**Results:**

Over median follow-up of 6.8 years, 209,991 of 3,156,863 participants (6.7%) died. The risk of death increased with increasing RDW percentile. After adjustment, and compared to RDW in the 25^th^ to 75^th^ percentiles, the risk of death was lower for participants in the <25^th^ percentiles but higher for participants in the 75^th^-95^th^ percentiles (HR 1.42, 95% CI 1.40,1.43), the 95^th^-99^th^ percentiles (HR 1.86, 95% CI 1.83,1.89) and the >99^th^ percentile (HR 2.18, 95% CI 2.12,2.23). Similar results were observed for MI, stroke/TIA, incident cancer, hospitalization and LTC placement, but no association was found between RDW and ESRD. Findings were generally similar for SD-RBC, except that all associations tended to be stronger than for RDW, and both lower and higher values of SD-RBC were independently associated with ESRD.

**Conclusion and relevance:**

RDW and SD-RBC may be useful as prognostic markers for people in the general population, especially for outcomes related to chronic illness. SD-RBC may be superior to RDW.

## Introduction

Routine laboratory data abounds in clinical care, generated using standardized assays that are widely available for diagnostic purposes. However, despite the ubiquitous nature of this data it is rarely exploited for prognostic purposes at the point of care. One such laboratory measure, the red cell distribution width (RDW), is calculated as the quotient of the standard deviation of the red blood cell size (SD-RBC) to the mean corpuscular volume (MCV), and reflects the extent of heterogeneity in the size of circulating erythrocytes.[1] RDW is reported automatically by most clinical laboratories as part of the complete blood count, and can be used to inform the etiology of anemia.[2] Previous work suggests that RDW may be useful as a prognostic biomarker, with higher RDW within the normal range independently associated with multiple cardiovascular outcomes including myocardial infarction, heart failure, stroke, atrial fibrillation and peripheral vascular disease.[3–7] The explanation for these associations is unclear but may relate to higher levels of circulating proinflammatory substances,[8] which both impair erythropoiesis and predispose to (or reflect the presence of) vascular disease.

Most prior studies examining RDW as a prognostic marker have been done in selected populations such as those with prior myocardial infarction[6] or known heart failure,[9] and most have focused on cardiovascular outcomes. Few studies have evaluated whether RDW is associated with adverse outcomes in the general population, and of those most have focused exclusively on cardiovascular outcomes. In addition, limited statistical power has compromised the ability of most prior studies to comprehensively adjust for potential confounders. Finally, whether SD-RBC is also associated with adverse outcomes other than those related to vascular disease (and the predictive power of SD-RBC vs RDW) has not been previously investigated.

We used a large population-based cohort to evaluate the independent association between RDW, SD-RBC and a range of clinical outcomes, including all-cause death, myocardial infarction, stroke or transient ischemic attack, incident kidney failure and incident solid malignancy. Because they are important to patients, we also considered placement in a long-term care (LTC) facility and all-cause hospitalization. We hypothesized that higher RDW at baseline would be independently associated with these clinical outcomes, and that higher SD-RBC would be more prognostically important than RDW. An important secondary objective was to assess for potential effect modifiers of any associations observed between RDW or SD-RBC and the adverse clinical outcomes.

## Methods

This retrospective population-based cohort study is reported according to the STROBE guidelines.[10] The institutional review boards at the Universities of Alberta (Pro00053469) and Calgary (REB14-0884) approved this study. The data were partially anonymized before we received them: data of birth and postal code remained in the dataset. The data custodian waived the requirement for informed consent.

### Data sources and cohort

We used the Alberta Kidney Disease Network database, which incorporates data from Alberta Health (AH; the provincial health ministry) including physician claims, hospitalizations and ambulatory care utilization; and the clinical laboratories in Alberta, Canada. This database has been widely used[11–13] because of its population-based coverage of a geographically defined area, including demographic characteristics, health services utilization, and clinical outcomes. Additional information on the database is available elsewhere, including the validation of selected data elements and the standardization and calibration of serum creatinine assays.[14] All adults 18 years of age and older registered with AH were included in the database; all Alberta residents are eligible for insurance coverage by AH and >99% participate in coverage. The database was used to assemble a cohort of adults who resided in Alberta, Canada between May 2003 and December 2016 with RDW and MCV measurements. We followed participants from May 2003, their first measure of RDW and MCV (baseline date) until death, out-migration or study end (March 2017), whichever was earlier.

### Red cell distribution width and other laboratory test values

To minimize effects of acute illness on hematological parameters, only outpatient laboratory results were included. We analyzed all of the following outpatient results from participants during the study period: RDW, hemoglobin, MCV, serum ferritin, white blood cell count, total and LDL cholesterol, (serum) albumin, estimated glomerular filtration rate (eGFR), albuminuria, and (high-sensitivity) c-reactive protein (CRP). We calculated SD-RBC from the product of RDW and MCV. The eGFR was estimated using the Chronic Kidney Disease Epidemiology equation. Albuminuria was measured using the albumin:creatinine ratio (ACR), the protein:creatinine ratio (PCR), and the urine dipstick. PCR was used when ACR was not available, and dipstick results were used when PCR was not available. Measurements were categorized as in prior work as follows: missing, none/mild (ACR <3 mg/mmol, PCR <15 mg/mmol, dipstick negative/trace), moderate (ACR 3–30 mg/mmol, PCR 15–50 mg/mmol, dipstick 1+), severe (ACR 31–220 mg/mmol, PCR 51–350 mg/mmol, dipstick 2+ and 3+), and nephrotic (ACR >220 mg/mmol, PCR >350 mg/mmol, dipstick ≥4+). Means (median for albuminuria) of the laboratory measurements within the first year of follow-up were used in the analyses. If no values were available from the first year, then values from subsequent years were imputed (first-value carried backwards).

### Outcomes

Clinical outcomes were time to all-cause death, first myocardial infarction,[15,16] first stroke or transient ischemic attack,[15,17] placement into long-term care (LTC), progression to renal replacement therapy (RRT; initiation of chronic dialysis or pre-emptive kidney transplantation), first hospitalization during follow-up, and first diagnosis of a solid malignancy[15,18,19] (i.e., excluding leukemias and lymphomas) during follow-up. LTC placement was defined by discharge to a private or public LTC facility following a hospital admission (for those not previously in a LTC facility) or 2 claims (at least 30 days apart) made from an LTC facility. RRT was determined using data from the Northern and Southern Alberta Renal programs. Participants with myocardial infarctions prior to baseline were excluded from analyses of myocardial infarctions during follow-up. Analyses of stroke, placements into LTC, progression to RRT and solid malignancy were similarly treated.

### Demographic characteristics

Demographic characteristics were captured from the Alberta Health registry: age, sex, Indigenous status, social assistance, and postal code of residence. Age was categorized as follows: 18–39, 40–64, 65–79, and ≥80 years. Rural residence location was determined from the postal code using the Statistic’s Canada Postal Code Conversion File (www.statcan.ca).

### Comorbidities

Comorbidities were defined using a previously published framework with 29 validated algorithms as applied to physician claims data, each of which had positive predictive values ≥70% as compared to a gold standard measure such as chart review.[15] Comorbidities included alcohol misuse, asthma, atrial fibrillation, lymphoma, non-metastatic cancer (breast, cervical, colorectal, pulmonary, and prostate cancer), metastatic cancer, chronic heart failure, chronic pain, chronic obstructive pulmonary disease, chronic hepatitis B, cirrhosis, severe constipation, dementia, depression, diabetes, epilepsy, hypertension, hypothyroidism, inflammatory bowel disease, irritable bowel syndrome, multiple sclerosis, myocardial infarction, Parkinson’s disease, peptic ulcer disease, peripheral vascular disease, psoriasis, rheumatoid arthritis, schizophrenia, and stroke or transient ischemic attack. We also considered chronic kidney disease as a 30th condition, which was defined by mean annual eGFR below 60 mL/min per 1.73 m^2^ or the presence of albuminuria (albumin:creatinine ratio ≥30 mg/g, protein:creatinine ratio ≥150 mg/g or dipstick proteinuria ≥trace). Each participant was classified with respect to the presence or absence of these 30 chronic conditions (lookback extended as far as April 1994 where records were available).[20] Detailed methods for classifying comorbidity status and the specific algorithms used are found elsewhere.[15]

### Statistical analyses

We did analyses with Stata MP 15·1 (www.stata.com) and reported unadjusted and age-sex adjusted baseline descriptive statistics as counts and percentages. We used Cox regression to determine the associations between baseline RDW percentiles (<1, 1–5, 5–25, 25–75, 75–95, 95–99, >99) and the first occurrence of each clinical outcomes during follow-up. We present four adjusted models: 1) adjustment for demographics (age, sex, Indigenous status, social assistance and rural status); 2) adjustment for demographics and all 30 baseline morbidities; 3) adjustment for demographics, morbidities, and baseline hemoglobin, WBC, and eGFR; and in a sensitivity analysis 4) adjustment for demographic characteristics, morbidities, and baseline hemoglobin, WBC, eGFR, albuminuria, and serum albumin. We determined that the proportional hazard assumptions were satisfied by examining plots of the log-negative-log of within-group survivorship probabilities versus log-time. Potential modifiers of the association between RDW percentiles and death were explored using interaction terms in model #4: age (≥65 years vs <65 years), sex (male vs female), diabetes, chronic heart failure, coronary artery disease, chronic kidney disease, anemia, and MCV above or below the median of 90 fL. In this analysis, coronary artery disease was defined by a history of myocardial infarction,[15,16] percutaneous coronary intervention (ICD-9 procedures codes: 36.01, 36.02, 36.05, 36.06, and CCI 1.IJ.50, 1.IJ.57.GQ, 1.IL.35) and coronary artery bypass grafting (ICD-9 procedures codes: 36.1, 36.2, and CCI 1.IJ.76). The models with and without the interaction terms were compared using the likelihood ratio test. Additionally we did a sensitivity analysis examining the relation between RDW and the risk of all-cause death, using additional categories for RDW (<0.01, 0.01–0.1, 0.1–1, 1–5, 5–25, 25–75, 75–95, 95–99, 99–99.9, 99.9–99.99, >99.99). Because the decision to initiate dialysis or receive a kidney transplantation is potentially subjective, we did a sensitivity analysis that considered the more objective outcome of sustained eGFR <15 mL/min*1·73m^2^ in addition to the initiation of RRT. Sustained eGFR <15 mL/min*1·73m^2^ was defined as the first sequence of eGFR values <15 mL/min*1.73m^2^ (with 2 values in the sequence at least 90 days apart). In further sensitivity analysis we adjusted Model 3 with the quadratic transformation of MCV, as MCV has a concave association with risk. These analyses were repeated for SD-RBC and compared with the results for RDW using the log-likelihood as the models had the same number of parameters. The threshold *p* for statistical significance was set at 0.05.

## Results

### Characteristics of study participants

Participant flow is shown in S1 Fig. Of 4,858,314 potential participants, 1,701,451 were excluded because they had no RDW and MCV measurements, leaving 3,156,863 participants. Participants with higher RDW values were older and more likely to be female than those with lower values. After adjustment for age and sex, participants with higher RDW were more likely to be Indigenous, receive social assistance, reside in an urban area, and have more morbidity (Table 1). The reference range for RDW <15.6%; the upper reference limit for RDW coincided with the 96^th^ percentile in the study population. Anemia and low MCV were highly prevalent in participants with RDW values exceeding the 95^th^ percentile. Participants with both low and high values of RDW were more likely to have abnormal laboratory values, although abnormalities appeared more common in participants with higher RDW. Similar observations were noted for participant with abnormal SD-RBC (S1 Table).

**Table 1:** Age and sex adjusted baseline demographics and clinical characteristics by RDW percentiles (N = 3,156,863).

Percentiles	<1	1–5	5–25	25–50	50–75	75–95	95–99	>99
Minimum value	8.0	11.4	11.9	12.6	13.1	13.7	15.3	18.2
N	37,439	122,373	544,697	808,056	903,583	576,637	132,327	31,751
Indigenous	1.7	2.0	1.9	2.0	2.5	4.3	6.9	9.9
Social assistance	2.0	1.8	1.9	2.3	3.0	4.5	6.1	6.7
Rural	22.3	18.7	12.8	9.3	8.2	8.8	9.8	11.4
Obesity	10.2	10.6	10.5	11.0	12.7	16.4	17.1	14.3
Albumin, g/L								
<35	2.4	1.9	1.8	2.0	2.5	4.3	9.9	15.1
>50	0.1	0.2	0.2	0.1	0.1	0.1	0.1	0.1
eGFR, mL/min*1.73m^2^								
>120	4.3	3.8	3.8	4.0	4.3	5.7	8.4	7.5
<60	10.1	10.1	9.9	9.9	10.4	11.6	14.2	18.4
Hemoglobin, g/L								
<120 F, <135 M	2.4	2.0	2.3	2.8	4.2	11.0	44.6	78.6
>160 F, >175 M	2.8	1.8	1.3	1.1	1.0	1.1	0.7	0.3
MCV, fL								
<80	0.2	0.1	0.1	0.3	0.7	5.6	34.7	59.9
>105	0.2	0.1	0.1	0.1	0.2	0.4	1.3	1.8
Proteinuria								
Severe/nephrotic	1.2	1.1	0.9	0.9	1.0	1.6	2.9	3.0
Moderate	4.4	3.7	3.3	3.2	3.4	4.4	6.1	6.8
WBC, X10^9^/L								
<4	3.4	3.2	3.1	2.9	2.6	2.8	4.0	5.7
>11	3.9	3.4	3.1	3.3	4.1	5.6	6.6	8.2
Morbidity								
Alcohol misuse	2.1	1.7	1.5	1.6	2.0	3.6	6.6	9.8
Asthma	1.8	1.5	1.5	1.5	1.7	2.3	2.9	3.1
AF	1.4	1.2	1.2	1.3	1.6	2.7	4.7	5.1
Lymphoma	0.2	0.1	0.1	0.1	0.2	0.4	1.5	2.5
Metastatic cancer	0.4	0.3	0.4	0.4	0.5	1.0	3.8	7.3
Single site cancer	1.5	1.4	1.5	1.6	1.7	2.0	2.8	3.7
CHF	1.7	1.3	1.3	1.3	1.7	3.1	7.0	7.6
CKD	11.5	10.7	10.9	11.3	12.3	16.0	21.5	21.4
Chronic pain	18.6	17.3	16.2	15.4	15.1	16.2	16.9	15.7
Chronic pulmonary	5.9	5.3	5.2	5.3	5.9	7.9	10.8	11.3
HBV	0.0	0.0	0.1	0.1	0.1	0.1	0.2	0.3
Cirrhosis	0.1	0.0	0.0	0.0	0.1	0.2	1.4	2.9
Severe constipation	1.2	0.9	0.9	0.8	0.9	1.3	2.3	3.1
Dementia	0.7	0.7	0.7	0.8	1.0	1.4	2.2	2.3
Depression	9.7	9.6	10.3	10.8	11.4	12.7	13.8	13.9
Diabetes	6.1	6.1	6.4	6.5	6.9	9.4	12.6	10.5
Epilepsy	1.2	1.1	1.1	1.2	1.3	1.6	2.2	2.6
Hypertension	18.7	18.7	19.1	19.5	20.6	23.8	26.3	23.6
Hypothyroid	5.1	5.3	5.7	5.9	6.2	7.0	7.3	6.4
IBD	0.6	0.5	0.5	0.6	0.7	1.3	3.0	4.9
IBS	1.4	1.3	1.4	1.4	1.3	1.3	1.3	1.4
MS	0.5	0.4	0.5	0.5	0.5	0.6	0.7	0.7
AMI	1.0	1.0	0.9	1.0	1.1	1.5	2.1	1.9
Parkinson’s	0.4	0.3	0.3	0.3	0.3	0.4	0.5	0.4
PUD	0.1	0.1	0.1	0.1	0.1	0.2	0.8	1.6
PAD	0.4	0.3	0.3	0.3	0.5	0.8	1.6	1.8
Psoriasis	0.3	0.3	0.4	0.4	0.4	0.5	0.6	0.6
Rheumatoid arthritis	0.7	0.7	0.8	0.8	1.0	1.8	3.1	2.9
Schizophrenia	0.5	0.5	0.6	0.7	0.9	1.3	1.4	1.4
Stroke or TIA	2.7	2.6	2.6	2.8	3.0	3.9	5.4	5.7

AFIB atrial fibrillation, CHF chronic heart failure, CKD chronic kidney disease, CVD cardiovascular event, eGFR estimated glomerular filtration rate, ESRD end-stage renal disease (initiation of renal replacement therapy), HBV viral hepatitis B, IBD inflammatory bowel disease, IBS irritable bowel syndrome, LTC long-term care, MCV mean corpuscular volume, MS multiple sclerosis, PAD peripheral arterial disease, PUD peptic ulcer disease, RDW red cell distribution width, TIA transient ischemic attack, WBC white blood count.

The table shows percentages except for N, which is a count. Social assistance and rural status could not be determined in 162,622 participants (5.2%) due to missing postal codes in the Alberta Health registry, obesity status could not be determined in 640,405 participants (20.3%) because they had not had a previous procedure, and a number of laboratory values had not been measured in standard of care practice—albumin in 1,623,410 participants (51.4%), eGFR in 212,922 participants (6.7%), hemoglobin in 22,767 participants (0.7%), proteinuria 473,299 participants (15.0%), and WBC in 169 participants (<0.1%).

Participants were followed for a median of 6.8 years (range 1 day to 13.9 years). There were 209,991 deaths (6.7%); 208,786 participants had at least one stroke or TIA (6.6%), 154,637 had at least one incident solid malignancy (4.9%), 101,358 were newly placed into long-term care (3.2%), 71,613 had at least one myocardial infarction (2.3%), 44,315 were hospitalized at least once (1.4%), and 9,615 initiated renal replacement therapy (0.3%).

### Mortality by RDW

**Fig 1:**
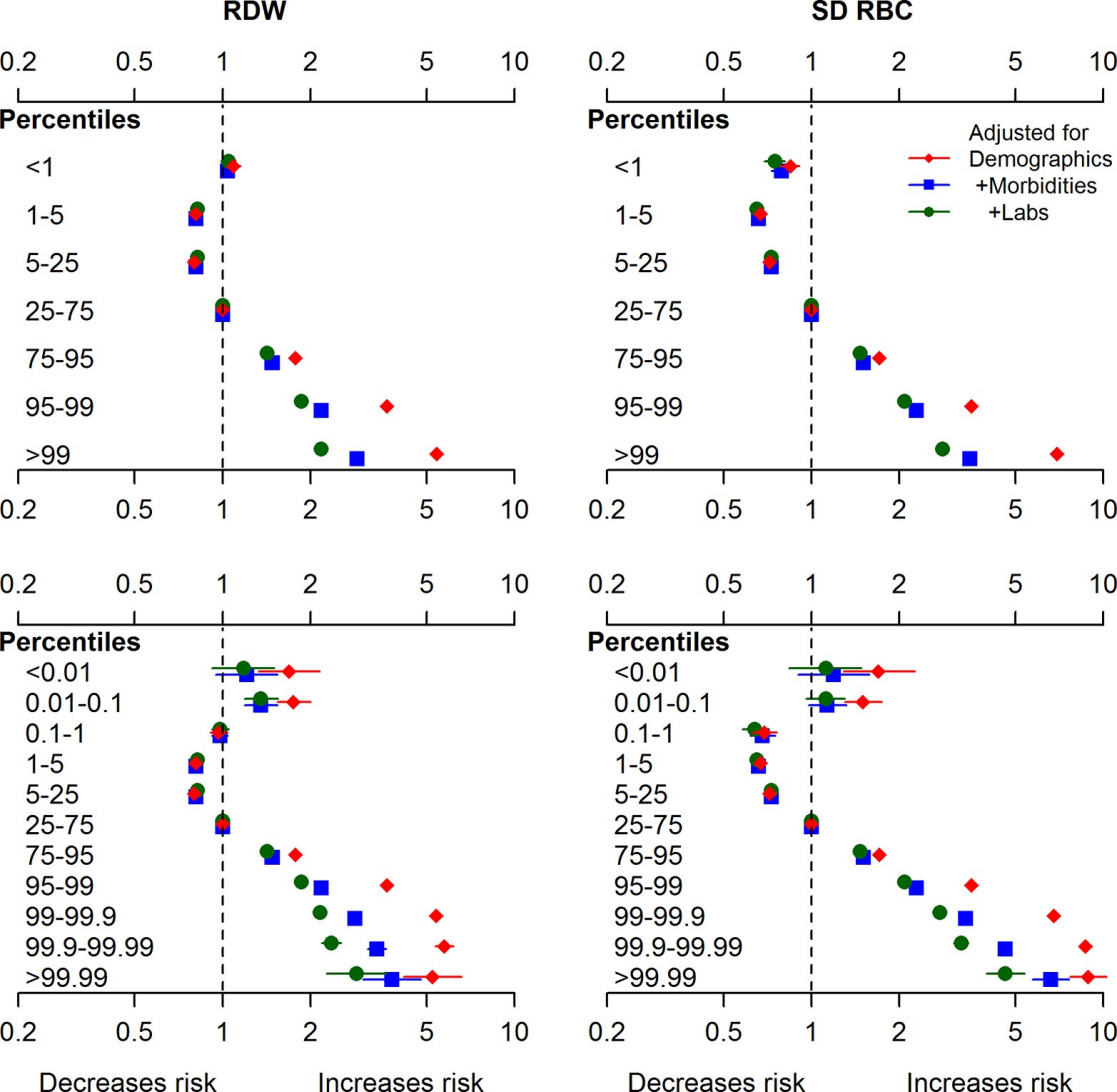
All-cause mortality by RDW and SD-RBC percentiles. eGFR estimated glomerular filtration rate, RDW red cell distribution width, SD-RBC standard deviation of red blood cell size, WBC white blood cells. Hazard ratios with 95% confidence intervals are reported for: 7 RDW percentile bins (top left), 7 SD-RBC percentile bins (top right), 11 RDW percentile bins (bottom left), and 11 SD-RBC percentile bins (bottom right). The first model (red diamonds) is adjusted for demographics: age, sex, Indigenous status, social assistance and rural status. The second model (blue squares) is adjusted for demographics and all 30 baseline morbidities. The third model (green circles) is adjusted for demographics, morbidities, and baseline hemoglobin, WBC, and eGFR.

With the exception of participants in the <1^st^ percentile, risk of death increased with increasing RDW percentile (Table 2). In the model adjusted for demographic characteristics only, compared to participants in the 25^th^ to 75^th^ percentiles, the risk of death was significantly lower for participants in the 1^st^ to 5^th^ percentiles (HR 0.81, 95% CI 0.78–0.84), and the 5^th^ to 25^th^ percentiles (HR 0.80, 95% CI 0.78,0.81) but higher for participants in the 75^th^ to 95^th^ percentiles (HR 1.78, 95% CI 1.76–1.80), the 95^th^ to 99^th^ percentiles (HR 3.66, 95% CI 3.61–3.71) and the >99^th^ percentile (HR 5.43, 95% CI 5.31–5.55). This pattern of relatively lower risk below the 25^th^ percentile and relatively higher risk above the 75^th^ percentile was consistent with increasing adjustment (Table 2), although the strength of the associations was attenuated. Results were again consistent in the sensitivity analyses that used 11 rather than 7 categories for RDW (Fig 1).

**Table 2:** Clinical outcomes associated with baseline RDW percentiles.

Percentiles	Death	MI	Stroke/TIA	LTC	ESRD	ESRD or sustained eGFR <15 mL/min*1.73m^2^	Hospitalization	Cancer
*Model 1*: *adjusted for demographic characteristics*								
N	3,156,863	3,123,591	3,070,812	3,153,179	3,153,179	3,152,079	3,155,971	3,105,540
<1	1.09 (1.03,1.15)	1.16 (1.05,1.29)	1.00 (0.94,1.07)	1.00 (0.91,1.10)	1.00 (0.72,1.38)	0.97 (0.73,1.29)	0.98 (0.87,1.10)	0.94 (0.88,1.00)
1–5	0.81 (0.78,0.84)	0.92 (0.86,0.98)	0.86 (0.83,0.90)	0.76 (0.71,0.80)	0.73 (0.59,0.90)	0.71 (0.59,0.86)	0.85 (0.79,0.91)	0.88 (0.85,0.92)
5–25	0.80 (0.78,0.81)	0.90 (0.87,0.92)	0.89 (0.88,0.91)	0.83 (0.81,0.86)	0.83 (0.76,0.92)	0.83 (0.76,0.90)	0.80 (0.77,0.83)	0.88 (0.87,0.90)
25–75	1.00	1.00	1.00	1.00	1.00	1.00	1.00	1.00
75–95	1.78 (1.76,1.80)	1.24 (1.21,1.28)	1.28 (1.27,1.30)	1.47 (1.44,1.49)	2.69 (2.53,2.86)	2.35 (2.23,2.48)	1.90 (1.85,1.94)	1.37 (1.36,1.39)
95–99	3.66 (3.61,3.71)	1.49 (1.42,1.55)	1.66 (1.62,1.70)	2.28 (2.23,2.34)	7.03 (6.51,7.59)	5.19 (4.84,5.56)	4.67 (4.54,4.81)	2.25 (2.20,2.30)
>99	5.43 (5.31,5.55)	1.40 (1.27,1.55)	1.79 (1.71,1.87)	2.77 (2.64,2.91)	4.82 (4.07,5.70)	3.99 (3.43,4.64)	8.02 (7.70,8.36)	3.26 (3.15,3.39)
*Model 2*: *adjusted for demographic characteristics and morbidities*								
N	3,156,863	3,123,591	3,070,812	3,153,179	3,153,179	3,152,079	3,155,971	3,105,540
<1	1.04 (0.99,1.10)	1.19 (1.08,1.32)	1.00 (0.94,1.07)	0.97 (0.88,1.06)	1.06 (0.76,1.46)	1.03 (0.77,1.37)	0.94 (0.83,1.05)	0.95 (0.89,1.01)
1–5	0.81 (0.79,0.85)	0.96 (0.90,1.03)	0.89 (0.86,0.92)	0.78 (0.73,0.83)	0.82 (0.66,1.01)	0.79 (0.66,0.96)	0.83 (0.78,0.90)	0.90 (0.87,0.93)
5–25	0.81 (0.80,0.82)	0.93 (0.90,0.96)	0.91 (0.90,0.93)	0.86 (0.84,0.89)	0.90 (0.82,0.99)	0.89 (0.82,0.97)	0.79 (0.76,0.82)	0.90 (0.88,0.91)
25–75	1.00	1.00	1.00	1.00	1.00	1.00	1.00	1.00
75–95	1.48 (1.47,1.50)	1.07 (1.05,1.10)	1.13 (1.11,1.15)	1.24 (1.22,1.26)	1.94 (1.83,2.07)	1.75 (1.65,1.84)	1.60 (1.56,1.64)	1.29 (1.27,1.31)
95–99	2.18 (2.15,2.22)	1.07 (1.03,1.12)	1.24 (1.21,1.27)	1.51 (1.47,1.55)	3.63 (3.35,3.94)	2.82 (2.63,3.04)	2.50 (2.43,2.58)	1.94 (1.90,1.99)
>99	2.89 (2.82,2.95)	1.09 (0.98,1.20)	1.38 (1.31,1.44)	1.80 (1.72,1.89)	2.82 (2.38,3.35)	2.42 (2.08,2.82)	3.39 (3.24,3.54)	2.80 (2.70,2.91)
*Model 3*: *adjusted for demographic characteristics*, *morbidities and hemoglobin*, *WBC*, *and eGFR*								
N	2,943,201	2,910,403	2,859,216	2,939,535	2,939,535	2,938,443	2,942,551	2,893,214
<1	1.05 (0.99,1.11)	1.16 (1.05,1.29)	1.01 (0.95,1.08)	0.98 (0.89,1.07)	1.08 (0.78,1.50)	1.01 (0.76,1.35)	0.98 (0.86,1.11)	0.96 (0.91,1.03)
1–5	0.82 (0.79,0.86)	0.95 (0.89,1.01)	0.89 (0.85,0.92)	0.80 (0.75,0.85)	1.09 (0.88,1.34)	1.04 (0.86,1.25)	0.88 (0.81,0.95)	0.91 (0.88,0.94)
5–25	0.82 (0.80,0.83)	0.93 (0.90,0.96)	0.92 (0.90,0.93)	0.87 (0.85,0.90)	1.02 (0.92,1.12)	1.00 (0.92,1.09)	0.82 (0.79,0.85)	0.90 (0.89,0.92)
25–75	1.00	1.00	1.00	1.00	1.00	1.00	1.00	1.00
75–95	1.42 (1.40,1.43)	1.09 (1.07,1.12)	1.13 (1.11,1.14)	1.20 (1.18,1.22)	1.15 (1.07,1.22)	1.14 (1.08,1.20)	1.44 (1.40,1.48)	1.25 (1.23,1.27)
95–99	1.86 (1.83,1.89)	1.17 (1.11,1.23)	1.24 (1.21,1.27)	1.32 (1.28,1.35)	1.29 (1.19,1.41)	1.17 (1.08,1.26)	1.83 (1.77,1.90)	1.72 (1.68,1.76)
>99	2.18 (2.12,2.23)	1.30 (1.18,1.44)	1.39 (1.32,1.46)	1.41 (1.34,1.48)	1.05 (0.88,1.26)	0.98 (0.83,1.14)	1.94 (1.84,2.04)	2.24 (2.15,2.34)
*Sensitivity analysis–Model 4*: *adjusted for demographic characteristics*, *morbidities and hemoglobin*, *WBC*, *eGFR*, *albuminuria and serum albumin*								
N	1,517,246	1,495,955	1,459,742	1,513,669	1,513,669	1,512,647	1,516,902	1,481,238
<1	0.97 (0.90,1.04)	1.13 (0.99,1.29)	0.99 (0.91,1.07)	0.86 (0.77,0.96)	1.13 (0.82,1.56)	1.04 (0.78,1.39)	0.94 (0.77,1.15)	0.93 (0.86,1.00)
1–5	0.77 (0.74,0.81)	0.93 (0.86,1.01)	0.90 (0.86,0.94)	0.78 (0.73,0.84)	1.10 (0.89,1.36)	1.05 (0.86,1.26)	0.92 (0.82,1.03)	0.91 (0.88,0.95)
5–25	0.81 (0.80,0.83)	0.92 (0.88,0.96)	0.92 (0.90,0.94)	0.86 (0.84,0.89)	1.05 (0.95,1.16)	1.02 (0.94,1.12)	0.82 (0.77,0.87)	0.91 (0.89,0.93)
25–75	1.00	1.00	1.00	1.00	1.00	1.00	1.00	1.00
75–95	1.34 (1.32,1.36)	1.05 (1.02,1.09)	1.10 (1.08,1.12)	1.16 (1.14,1.18)	1.02 (0.95,1.08)	1.01 (0.96,1.07)	1.42 (1.37,1.47)	1.19 (1.17,1.21)
95–99	1.65 (1.62,1.68)	1.10 (1.04,1.16)	1.18 (1.15,1.21)	1.23 (1.20,1.27)	1.12 (1.03,1.22)	1.02 (0.95,1.11)	1.76 (1.68,1.85)	1.59 (1.55,1.63)
>99	1.93 (1.87,1.98)	1.31 (1.17,1.46)	1.35 (1.28,1.43)	1.29 (1.22,1.37)	0.77 (0.65,0.93)	0.74 (0.63,0.86)	1.86 (1.74,1.98)	2.07 (1.98,2.16)

eGFR estimated glomerular filtration rate, ESRD end-stage renal disease (initiation of renal replacement therapy), LTC long-term care, MI myocardial infarction, RDW red cell distribution width, TIA transient ischemic attack, WBC white blood counts.

Hazard ratios with 95% confidence intervals are reported. The first model is adjusted for demographics: age, sex, Indigenous status, social assistance and rural status. The second model is adjusted for demographics and all 30 baseline morbidities. The third model is adjusted for demographics, morbidities, and baseline hemoglobin, WBC, and eGFR. The fourth model is adjusted for demographics, morbidities, and baseline hemoglobin, WBC, eGFR, albuminuria, and serum albumin–as a sensitivity analysis.

### Mortality by SD-RBC

Qualitatively, the association between SD-RBC and mortality was similar to that between RDW and mortality (Fig 1; S2 Table). However, the magnitude of the hazard ratio associated with a particular percentile tended to be greater for SD-RBC than for RDW, and the log-likelihood for the former was substantially less negative than the latter (-2,605,690 vs -2,609,914)–both suggesting that the predictive power of SD-RBC was greater than that of RDW.

**Fig 2:**
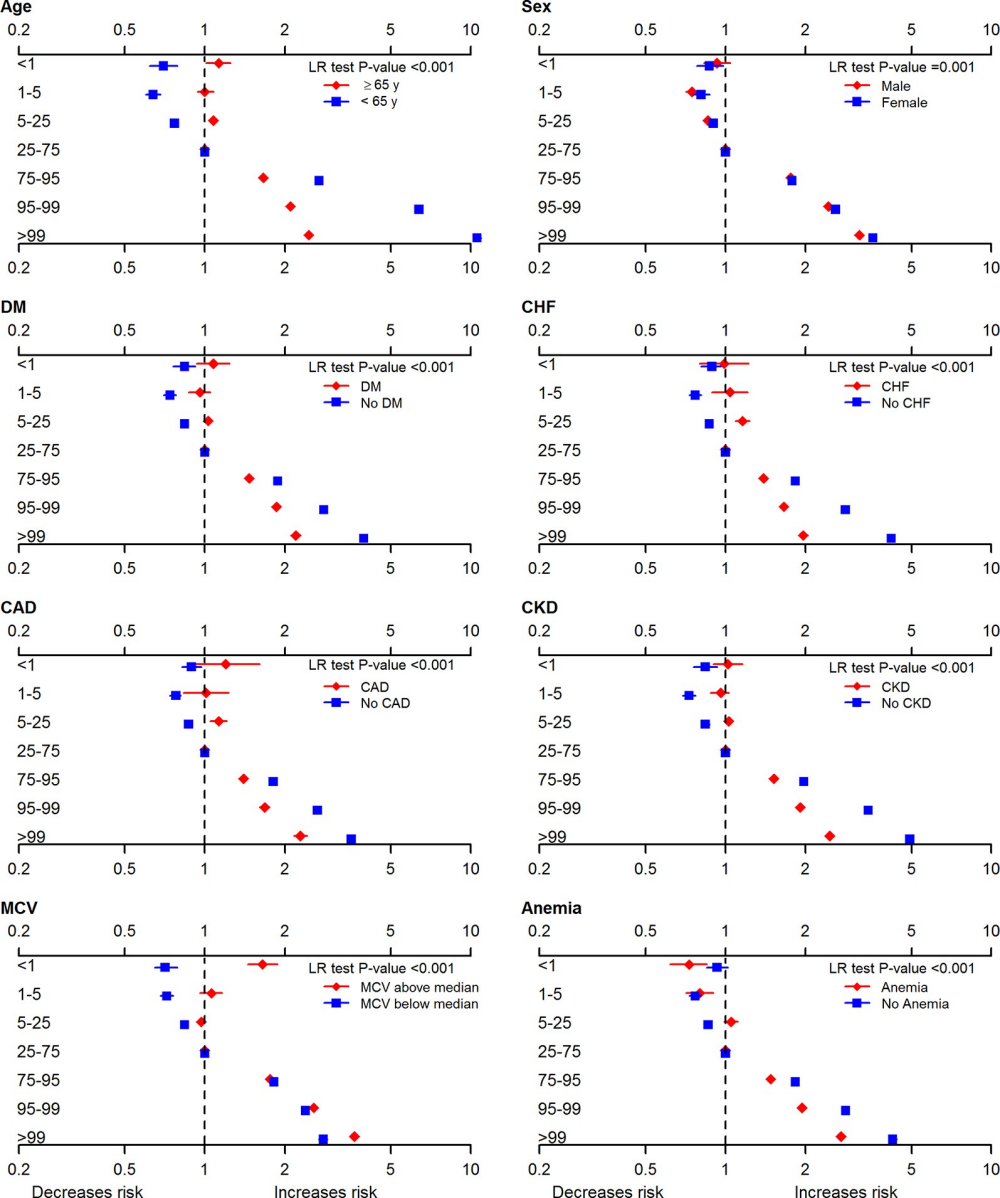
All-cause mortality by SD-RBC percentiles, stratified by subgroup. CAD coronary artery disease, CKD chronic kidney disease, DM diabetes mellitus, eGFR estimates glomerular filtration rate, LR likelihood ratio, MCV mean corpuscular volume, SD-RBC standard deviation of red blood cell size, WBC white blood cells. Hazard ratios with 95% confidence intervals are reported for 7 SD-RBC percentile bins for following subgroups: age (≥65 years vs <65 years), sex, diabetes mellitus, chronic heart failure, coronary artery disease, chronic kidney disease, mean corpuscular volume (above vs below median of 90 fL), and anemia. The model is adjusted for demographics, morbidities, and baseline hemoglobin, WBC, and eGFR.

Tests for interaction suggested that the association between RDW and death and between SD-RBC and death were both significantly stronger in participants that were younger, female, had less comorbidity, and had higher MCV (Fig 2 and S2 Fig).

### Cancer, stroke/TIA, and myocardial infarction by RDW and SD-RBC

**Fig 3:**
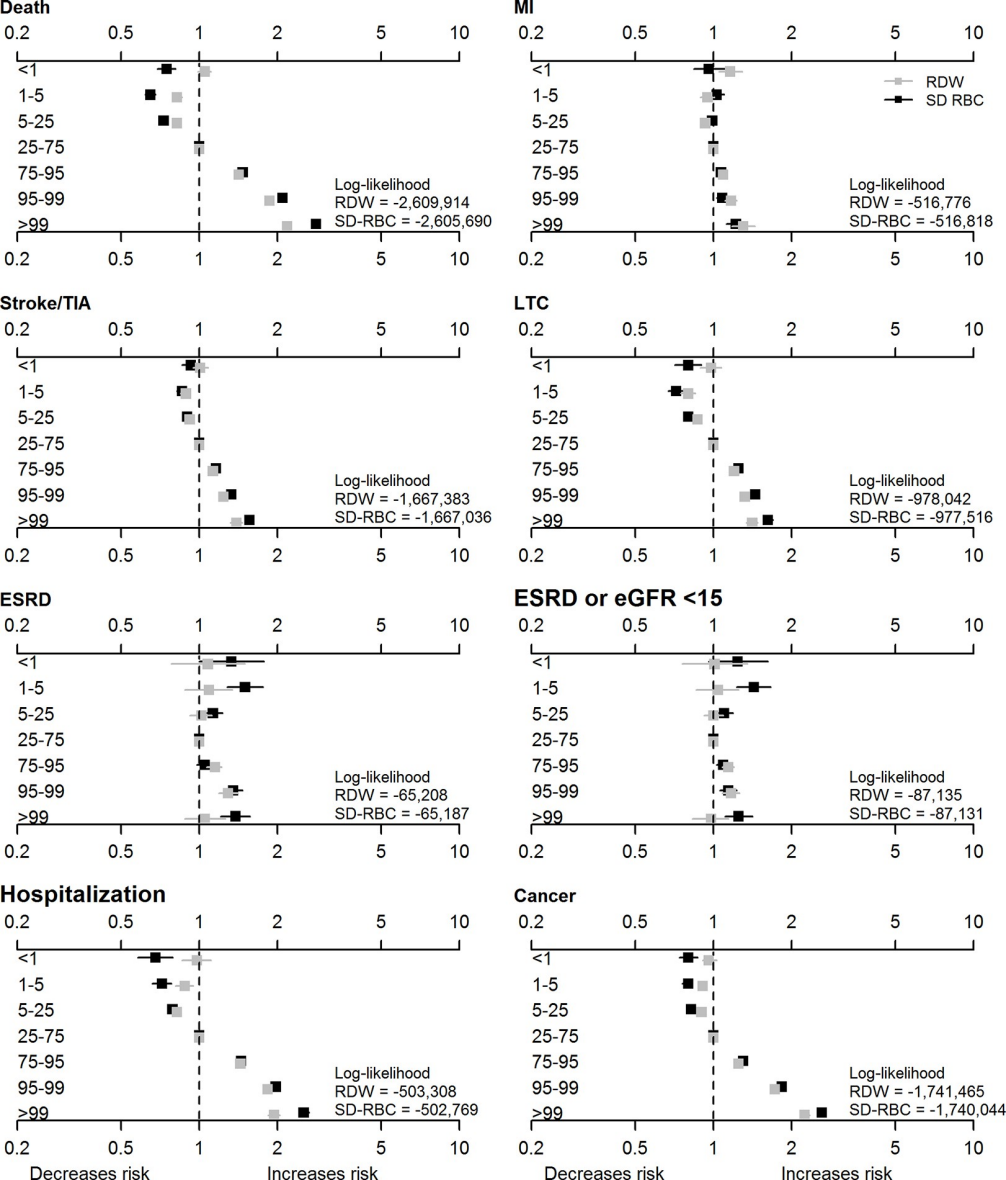
Clinical outcomes by RDW and SD-RBC percentiles. eGFR estimated glomerular filtration rate, ESRD end-stage renal disease (initiation of renal replacement therapy), LTC long-term care, MI myocardial infarction, RDW red cell distribution width, SD-RBC red blood cell standard deviation, TIA transient ischemic attack, WBC white blood counts. Hazard ratios with 95% confidence intervals are reported for 7 RDW (gray squares) and 7 SD-RBC (black squares) percentile bins. The models are adjusted for demographics, morbidities, and baseline hemoglobin, WBC, and eGFR.

After full adjustment, the associations of RDW with the risk of cancer, stroke/TIA and myocardial infarction were generally similar to the association with the risk of death (Table 2), although the magnitude of the excess risk appeared smaller for these other outcomes. Once again, the magnitude of the excess risk appeared larger and the corresponding log-likelihood values were more negative for associations related to SD-RBC than to RDW (S2 Table; Fig 3). The additional predictive power associated with SD-RDW appeared larger for cancer and stroke/TIA than for myocardial infarction.

### Hospitalization, long-term care placement and ESRD by RDW and SD-RBC

The likelihood of hospitalization and new long-term care placements also increased in parallel with both RDW (Table 2) and SD-RBC (S2 Table), and the magnitude of these associations again appeared stronger for SD-RBC (Fig 3), especially for hospitalization.

Although higher RDW was strongly associated with the likelihood of ESRD in the model that was adjusted solely for demographic characteristics (Table 2), the association was progressively attenuated with further adjustment for confounders, and was not observed in the fully adjusted model. Results for RDW were similar when we included sustained eGFR <15 mL/min*1.73m^2^ in addition to initiation of RRT. In contrast, both lower (<25^th^ percentile) and higher (≥95^th^ percentile) values of SD-RBC were associated with excess risk of ESRD in the fully adjusted model (S2 Table; Fig 3).

### Sensitivity analyses

Results were generally similar in the sensitivity analyses that additionally adjusted for WBC, eGFR, albuminuria and serum albumin, although the sample size was significantly smaller and the magnitude of the excess risk appeared lower than in the fully adjusted model (Table 2; S2 Table).

## Discussion

Consistent with our hypotheses, we found a strong and independent relation between RDW, SD-RBC and a range of clinical outcomes including all-cause mortality, stroke/TIA, myocardial infarction, all-cause hospitalization, placement in an LTC facility, and incident solid malignancy. Higher levels of both parameters tended to be associated with multiple potential confounders, as demonstrated by the progressive decreases in the strength of these associations with progressively more comprehensive statistical adjustment. After full adjustment, the magnitude of the excess risk associated with higher levels of RDW that remained within the normal range (e.g., 75-95^th^ percentile) varied from 30% for myocardial infarction to approximately 100% for all-cause death, hospitalization and cancer, as compared to values in the 25^th^-75^th^ percentile. Progressively higher levels of RDW were associated with additional increases in risk for all of these outcomes. The lowest risk was observed with values below the 25^th^ percentile, although there was no consistent evidence that even lower values (e.g., <5^th^ or <1^st^ percentile) were associated with further decreases in risk. In contrast, and contrary to our expectations, we found no independent association between higher levels of RDW and the risk of incident kidney failure, regardless of whether the latter was defined by solely by initiation of renal replacement or included the sustained occurrence of eGFR <15 mL/min/1.73m^2^.

Results for SD-RBC were generally similar to those for RDW, but significant and independent associations with SD-RBC outside the 25-75^th^ percentiles were observed for all outcomes including ESRD, were consistently larger than the corresponding associations with RDW, and extended for some outcomes (e.g. mortality; ESRD) to values below the first percentile. The exception was myocardial infarction, for which RDW appeared to have slightly better predictive power than SD-RBC. We cannot explain why RDW should be more strongly associated with myocardial infarction than SD-RBC, or why SD-RBC but not RDW should be associated with the risk of ESRD. We also found that several clinical characteristics modified the association between RDW, SD-RBC and adverse outcomes; both parameters were more strongly associated with excess risk among females, younger participants, and those with less comorbidity or higher MCV at baseline. On balance, these findings suggest that SD-RBC may be more useful as a prognostic marker than RDW for people in the general population.

Previous studies have evaluated the association between RDW and adverse outcomes in populations with coronary disease, heart failure, cerebrovascular disease, peripheral vascular disease, hypertension, and cancer[6,9,21–24]—and have considered a range of outcomes including clinical events (e.g., death, myocardial infarction) and surrogate markers (e.g., carotid atherosclerosis, ventricular filling pressures). Higher levels of RDW have been consistently associated with adverse risk profiles in the large majority of all such studies, although the mechanism is unclear. A few studies have been done in general population samples, although most were relatively small (n<30,000) and most tended to focus on all-cause mortality.[25–28] A notable exception was an Israel-based study of 225,006 people which showed that elevated RDW was associated with major cardiovascular events as well as all-cause mortality.[29] Our findings are similar to those from a recently published study done using 240,477 apparently healthy participants in the UK biobank,[30] which demonstrated that higher RDW was associated with a range of adverse outcomes including all-cause mortality, incident coronary disease, heart failure, peripheral vascular disease, atrial fibrillation, stroke, and cancer.

Our study extends this previous work by studying an unselected population-based cohort, evaluating a comprehensive list of clinically relevant outcomes, and adjusting for more than 40 potential confounders including a detailed panel of comorbidities, other hematological parameters (e.g., MCV, hemoglobin), and other plausible confounders such as eGFR, albuminuria, and serum albumin. We did not identify prior studies that examined the predictive power of SD-RDW.

Higher levels of RDW might be due to subacute inflammation[8] (accompanying, causing, or exacerbating vascular disease), disease-related nutritional deficiencies resulting in altered hematopoiesis,[31,32] or abnormal production and/or survival of circulating erythrocytes (perhaps reflecting occult systemic illness). However, we did not have data to evaluate these possibilities and thus these suggestions are speculative. Although identifying the mechanisms that underpin the associations reported herein may lead to further insights, we also found that RDW is independently associated with non-specific adverse outcomes such as hospitalization and placement in an LTC facility, suggesting that it may be useful as a summary biomarker of underlying chronic illness, rather than being pathophysiologically linked to a particular disease or diseases. Since RDW and potentially SD-RDW are both available at no added cost when complete blood counts are done, our findings suggest that these parameters are promising potential prognostic markers for use in clinical practice. The finding that higher values of RDW and SD-RBC carry more prognostic weight in some subgroups than in others may be a first step toward this goal.

Our study has several important strengths. We used population-based data from more than 3.1 million people from a geographically defined area served by a universal health care system. We used validated algorithms for ascertaining the presence or absence of a comprehensive panel of comorbidities and used these covariates to adjust for potential confounders in analyses that considered a broad range of clinically relevant outcomes. However, our study also has certain limitations that should be considered. First, as with all studies using administrative data, some misclassification is possible for both comorbidities and outcomes. However, any such misclassification should have been nondifferential, and is unlikely to account for the observed association between RDW, SD-RBC and the outcomes that we studied. Second, we studied people from a single Canadian province, although it seems unlikely that repeating the study elsewhere would lead to different conclusions. Third, more than 1.7 million Alberta residents were excluded from our study because they did not have the exposures of interest measured during the study period. Therefore, our findings can be safely generalized only to those who have complete blood counts measured as part of routine care. Finally, we only had access to data that were ordered for clinical purposes, and so did not have data on serum albumin or albuminuria for all participants, and did not have any data on inflammatory biomarkers. Although residual confounding by these characteristics is possible, the latter would arguably be on the causal pathway for the association between RDW or SD-RBC and adverse outcomes, and thus adjustment for these characteristics may not be appropriate in analyses for prognostic significance.

## Conclusions

In conclusion, RDW and SD-RBC were both independently associated with a range of clinical outcomes in a population-based cohort, including all-cause mortality, stroke/TIA, myocardial infarction, all-cause hospitalization, placement in an LTC facility, and incident solid malignancy. The associations were stronger in women, older participants, and in those with pre-existing conditions such as coronary disease, heart failure, and chronic kidney disease–and were consistently more robust for SD-RBC than for RDW. These findings suggest that one or both of these parameters may be useful as potential prognostic markers for people in the general population, especially for outcomes related to chronic illness.

## Supporting information

S1 Fig Participant flow diagram. AKDN Alberta Kidney Disease Network, MCV mean corpuscular volume, RDW red cell distribution width.
(TIF)

S2 Fig All-cause mortality by RDW percentiles, stratified by subgroup. CAD coronary artery disease, CKD chronic kidney disease, DM diabetes mellitus, LR likelihood ratio, MCV mean corpuscular volume, RDW red cell distribution width. Hazard ratios with 95% confidence intervals are reported for 7 RDW percentile bins for following subgroups: age (≥65 years vs <65 years), sex, diabetes mellitus, chronic heart failure, coronary artery disease, chronic kidney disease, mean corpuscular volume (above vs below median of 90 fL), and anemia. The model is adjusted for demographics, morbidities, and baseline hemoglobin, WBC, and eGFR.
(TIFF)

S1 Table Age and sex adjusted baseline demographics and clinical characteristics by SD-RBC percentiles (N = 3,156,863). AFIB atrial fibrillation, CHF chronic heart failure, CKD chronic kidney disease, CVD cardiovascular event, eGFR estimated glomerular filtration rate, ESRD end-stage renal disease (initiation of renal replacement therapy), HBV viral hepatitis B, IBD inflammatory bowel disease, IBS irritable bowel syndrome, LTC long-term care, MCV mean corpuscular volume, MS multiple sclerosis, PAD peripheral arterial disease, PUD peptic ulcer disease, RDW red cell distribution width, TIA transient ischemic attack, WBC white blood counts. The table shows percentages except for N, which is a count. Social assistance and rural status could not be determined in 162,622 participants (5.2%) due to missing postal codes in the Alberta Health registry, obesity status could not be determined in 640,405 participants (20.3%) because they had not had a previous procedure, and a number of laboratory values had not been measured in standard of care practice—albumin in 1,623,410 participants (51.4%), eGFR in 212,922 participants (6.7%), hemoglobin in 22,767 participants (0.7%), proteinuria 473,299 participants (15.0%), and WBC in 169 participants (<0.1%).
(DOCX)

S2 Table Clinical outcomes associated with baseline SD-RBC percentiles. eGFR estimated glomerular filtration rate, ESRD end-stage renal disease (initiation of renal replacement therapy), LTC long-term care, MI myocardial infarction, SD-RBC red blood cell standard deviation, TIA transient ischemic attack, WBC white blood counts. Hazard ratios with 95% confidence intervals are reported. The first model is adjusted for demographics: age, sex, Indigenous status, social assistance and rural status. The second model is adjusted for demographics and all 30 baseline morbidities. The third model is adjusted for demographics, morbidities, and baseline hemoglobin, WBC, and eGFR. The fourth model (sensitivity analysis) is adjusted for demographics, morbidities, and baseline hemoglobin, WBC, eGFR, albuminuria, and serum albumin.
(DOCX)

S1 Data Availability Statement (DOCX)
