# Supplementary figures and images for: Red cell distribution width associations with clinical outcomes: A population-based cohort study
Source: PLoS One. 2019 Mar 13;14(3):e0212374. doi: 10.1371/journal.pone.0212374 (PMC6415845; doi:10.1371/journal.pone.0212374)

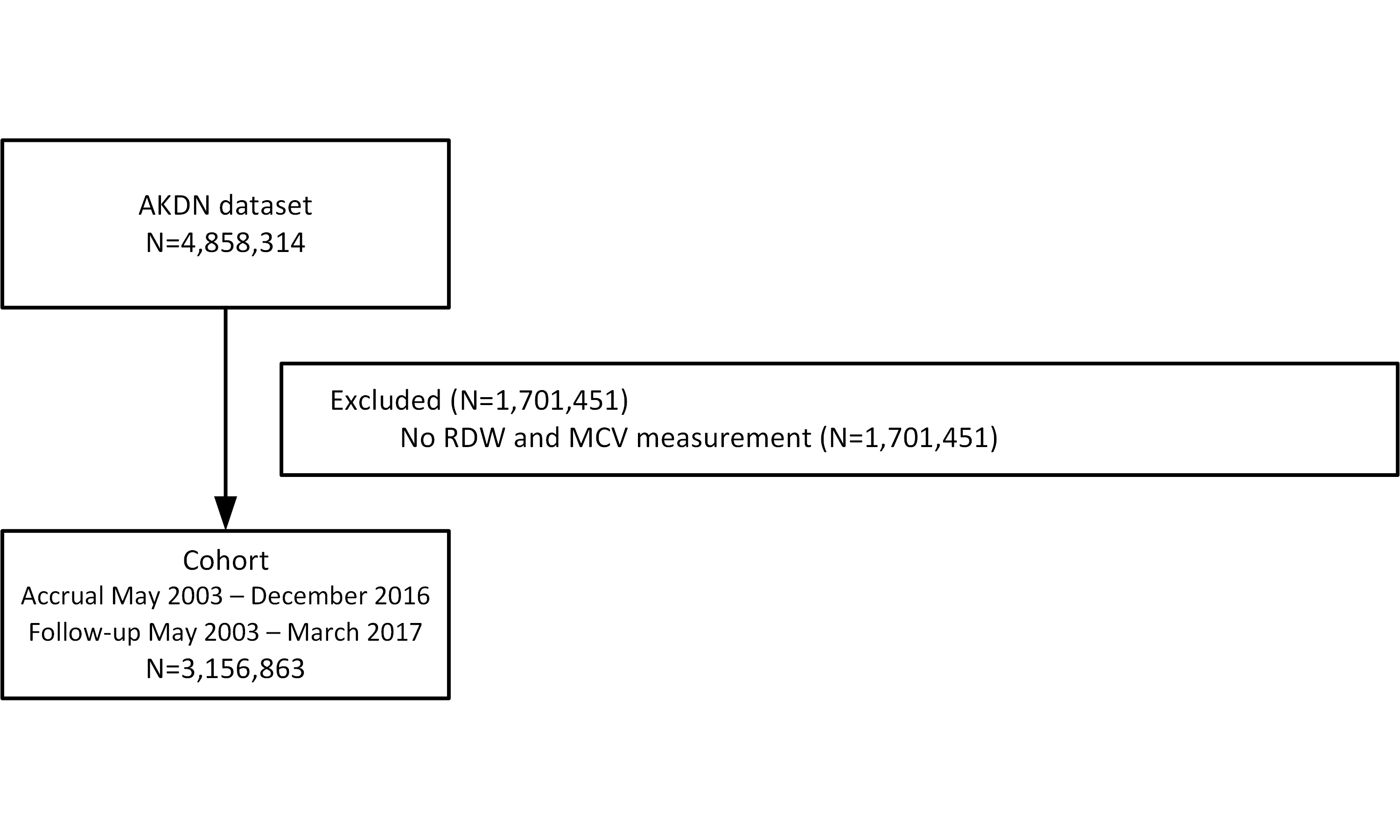

Supplement: S1 Fig — AKDN Alberta Kidney Disease Network, MCV mean corpuscular volume, RDW red cell distribution width. (TIF) [file pone.0212374.s001.tif]

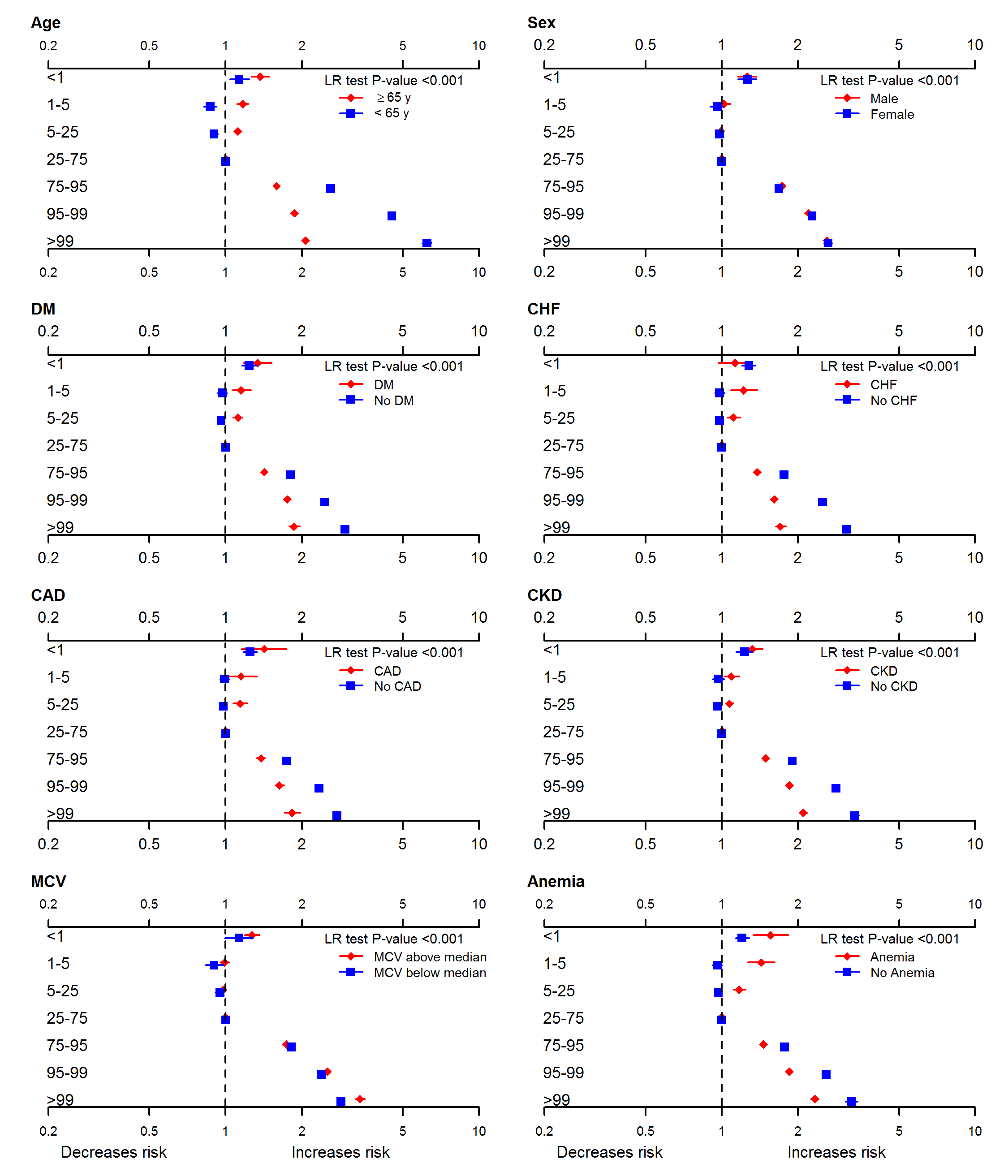

Supplement: S2 Fig — CAD coronary artery disease, CKD chronic kidney disease, DM diabetes mellitus, LR likelihood ratio, MCV mean corpuscular volume, RDW red cell distribution width. Hazard ratios with 95% confidence intervals are reported for 7 RDW percentile bins for following subgroups: age (≥65 years vs <65 years), sex, diabetes mellitus, chronic heart failure, coronary artery disease, chronic kidney disease, mean corpuscular volume (above vs below median of 90 fL), and anemia. The model is adjusted for demographics, morbidities, and baseline hemoglobin, WBC, and eGFR. (TIFF) [file pone.0212374.s002.tiff]
