# Supplementary material for: Red cell distribution width associations with clinical outcomes: A population-based cohort study
Source: PLoS One. 2019 Mar 13;14(3):e0212374. doi: 10.1371/journal.pone.0212374 (PMC6415845; doi:10.1371/journal.pone.0212374)
Supplement: S1 Table — AFIB atrial fibrillation, CHF chronic heart failure, CKD chronic kidney disease, CVD cardiovascular event, eGFR estimated glomerular filtration rate, ESRD end-stage renal disease (initiation of renal replacement therapy), HBV viral hepatitis B, IBD inflammatory bowel disease, IBS irritable bowel syndrome, LTC long-term care, MCV mean corpuscular volume, MS multiple sclerosis, PAD peripheral arterial disease, PUD peptic ulcer disease, RDW red cell distribution width, TIA transient ischemic attack, WBC white blood counts. The table shows percentages except for N, which is a count. Social assistance and rural status could not be determined in 162,622 participants (5.2%) due to missing postal codes in the Alberta Health registry, obesity status could not be determined in 640,405 participants (20.3%) because they had not had a previous procedure, and a number of laboratory values had not been measured in standard of care practice—albumin in 1,623,410 participants (51.4%), eGFR in 212,922 participants (6.7%), hemoglobin in 22,767 participants (0.7%), proteinuria 473,299 participants (15.0%), and WBC in 169 participants (<0.1%). (DOCX) [file pone.0212374.s003.docx]

| **Percentiles** | **<1** | **1-5** | **5-25** | **25-50** | **50-75** | **75-95** | **95-99** | **>99** |
| --- | --- | --- | --- | --- | --- | --- | --- | --- |
| **Minimum value** | **254** | **998** | **1,054** | **1,130** | **1,183** | **1,244** | **1,368** | **1,552** |
| N | 32,145 | 126,249 | 630,134 | 789,901 | 789,218 | 631,375 | 126,274 | 31,567 |
| Indigenous | 1.8 | 2.2 | 2.2 | 2.3 | 2.7 | 3.9 | 7.5 | 9.5 |
| Social assistance | 2.0 | 1.8 | 2.0 | 2.3 | 2.8 | 4.3 | 8.1 | 9.6 |
| Rural | 17.8 | 17.1 | 13.0 | 9.9 | 8.5 | 8.2 | 10.1 | 11.3 |
| Obesity | 11.3 | 12.6 | 12.7 | 12.2 | 12.4 | 13.0 | 14.1 | 12.1 |
| Albumin, g/L |  |  |  |  |  |  |  |  |
| <35 | 2.1 | 2.1 | 1.9 | 2.0 | 2.5 | 3.7 | 9.3 | 18.9 |
| >50 | 0.2 | 0.2 | 0.2 | 0.1 | 0.1 | 0.1 | 0.2 | 0.2 |
| eGFR, mL/min*1.73m^2^ |  |  |  |  |  |  |  |  |
| >120 | 4.3 | 3.6 | 3.9 | 4.0 | 4.4 | 5.1 | 7.9 | 10.4 |
| <60 | 12.3 | 11.4 | 10.7 | 10.2 | 10.2 | 10.4 | 12.8 | 17.3 |
| Hemoglobin, g/L |  |  |  |  |  |  |  |  |
| <120 F, <135 M | 15.5 | 6.9 | 4.0 | 4.1 | 5.3 | 9.5 | 28.9 | 59.6 |
| >160 F, >175 M | 1.6 | 1.2 | 1.0 | 1.0 | 1.1 | 1.3 | 1.8 | 1.0 |
| MCV, fL |  |  |  |  |  |  |  |  |
| <80 | 26.5 | 12.2 | 3.5 | 1.6 | 1.5 | 2.8 | 9.9 | 20.3 |
| >105 | 0.0 | 0.0 | 0.0 | 0.0 | 0.0 | 0.1 | 2.4 | 12.3 |
| Proteinuria |  |  |  |  |  |  |  |  |
| Severe/nephrotic | 1.2 | 1.3 | 1.0 | 0.9 | 1.0 | 1.3 | 2.7 | 3.7 |
| Moderate | 4.4 | 3.9 | 3.5 | 3.4 | 3.4 | 4.0 | 5.9 | 7.3 |
| WBC, X10^9^/L |  |  |  |  |  |  |  |  |
| <4 | 2.8 | 2.8 | 2.8 | 2.8 | 2.8 | 2.8 | 4.1 | 7.9 |
| >11 | 3.8 | 3.5 | 3.2 | 3.4 | 4.0 | 5.3 | 6.9 | 8.8 |
| Morbidity |  |  |  |  |  |  |  |  |
| Alcohol misuse | 1.3 | 1.0 | 1.1 | 1.4 | 1.9 | 3.8 | 9.9 | 16.0 |
| Asthma | 1.5 | 1.4 | 1.5 | 1.6 | 1.7 | 2.1 | 3.3 | 3.7 |
| AF | 1.1 | 1.1 | 1.2 | 1.3 | 1.6 | 2.3 | 4.4 | 5.9 |
| Lymphoma | 0.2 | 0.1 | 0.1 | 0.1 | 0.2 | 0.3 | 1.4 | 3.5 |
| Metastatic cancer | 0.5 | 0.4 | 0.4 | 0.4 | 0.5 | 0.9 | 3.1 | 8.3 |
| Single site cancer | 1.6 | 1.3 | 1.4 | 1.6 | 1.7 | 2.0 | 2.8 | 3.9 |
| CHF | 1.7 | 1.3 | 1.2 | 1.4 | 1.7 | 2.6 | 6.2 | 9.3 |
| CKD | 12.3 | 11.6 | 11.2 | 11.6 | 12.4 | 14.6 | 20.7 | 25.6 |
| Chronic pain | 16.8 | 16.6 | 16.3 | 15.6 | 15.1 | 15.6 | 18.3 | 18.0 |
| Chronic pulmonary | 5.1 | 5.1 | 5.1 | 5.2 | 5.8 | 7.5 | 12.0 | 14.3 |
| HBV | 0.1 | 0.1 | 0.0 | 0.1 | 0.1 | 0.1 | 0.2 | 0.3 |
| Cirrhosis | 0.0 | 0.0 | 0.0 | 0.0 | 0.0 | 0.1 | 1.2 | 4.9 |
| Severe constipation | 1.0 | 0.9 | 0.8 | 0.9 | 0.9 | 1.1 | 2.1 | 3.7 |
| Dementia | 0.7 | 0.6 | 0.7 | 0.7 | 0.9 | 1.3 | 2.2 | 2.8 |
| Depression | 8.2 | 8.4 | 9.7 | 10.5 | 11.3 | 13.2 | 17.3 | 18.7 |
| Diabetes | 9.1 | 8.4 | 7.7 | 7.2 | 7.0 | 7.4 | 9.8 | 10.9 |
| Epilepsy | 0.9 | 0.8 | 0.9 | 1.0 | 1.3 | 1.9 | 3.4 | 4.5 |
| Hypertension | 19.4 | 20.3 | 20.1 | 20.2 | 20.6 | 21.7 | 25.2 | 26.7 |
| Hypothyroid | 5.4 | 5.4 | 5.8 | 6.0 | 6.1 | 6.5 | 7.5 | 7.2 |
| IBD | 0.5 | 0.5 | 0.6 | 0.7 | 0.8 | 1.2 | 3.4 | 5.7 |
| IBS | 1.2 | 1.1 | 1.3 | 1.3 | 1.3 | 1.4 | 1.6 | 1.7 |
| MS | 0.3 | 0.4 | 0.4 | 0.5 | 0.5 | 0.6 | 0.8 | 1.0 |
| AMI | 0.9 | 0.8 | 0.9 | 0.9 | 1.1 | 1.4 | 2.1 | 2.2 |
| Parkinson’s | 0.3 | 0.2 | 0.3 | 0.3 | 0.3 | 0.4 | 0.5 | 0.5 |
| PUD | 0.1 | 0.1 | 0.1 | 0.1 | 0.1 | 0.2 | 0.7 | 1.6 |
| PAD | 0.3 | 0.3 | 0.3 | 0.3 | 0.4 | 0.7 | 1.6 | 2.4 |
| Psoriasis | 0.4 | 0.3 | 0.3 | 0.4 | 0.4 | 0.5 | 0.7 | 0.7 |
| Rheumatoid arthritis | 0.7 | 0.6 | 0.8 | 0.8 | 1.0 | 1.6 | 3.4 | 3.8 |
| Schizophrenia | 0.4 | 0.5 | 0.6 | 0.7 | 0.9 | 1.3 | 1.9 | 1.8 |
| Stroke or TIA | 2.5 | 2.4 | 2.6 | 2.7 | 3.0 | 3.7 | 5.6 | 7.2 |
