# Supplementary material for: Red cell distribution width associations with clinical outcomes: A population-based cohort study
Source: PLoS One. 2019 Mar 13;14(3):e0212374. doi: 10.1371/journal.pone.0212374 (PMC6415845; doi:10.1371/journal.pone.0212374)
Supplement: S2 Table — eGFR estimated glomerular filtration rate, ESRD end-stage renal disease (initiation of renal replacement therapy), LTC long-term care, MI myocardial infarction, SD-RBC red blood cell standard deviation, TIA transient ischemic attack, WBC white blood counts. Hazard ratios with 95% confidence intervals are reported. The first model is adjusted for demographics: age, sex, Indigenous status, social assistance and rural status. The second model is adjusted for demographics and all 30 baseline morbidities. The third model is adjusted for demographics, morbidities, and baseline hemoglobin, WBC, and eGFR. The fourth model (sensitivity analysis) is adjusted for demographics, morbidities, and baseline hemoglobin, WBC, eGFR, albuminuria, and serum albumin. (DOCX) [file pone.0212374.s004.docx]

| **Percentiles** | **Death** | **MI** | **Stroke/TIA** | **LTC** | **ESRD** | **Hospitalization** | **Cancer** |
| --- | --- | --- | --- | --- | --- | --- | --- |
| *Model 1: adjusted for demographic characteristics* | | | | | | | |
| N | 3,156,863 | 3,123,591 | 3,070,812 | 3,153,179 | 3,153,179 | 3,155,971 | 3,105,540 |
| <1 | 0.85 (0.78,0.91) | 0.92 (0.80,1.06) | 0.90 (0.84,0.97) | 0.87 (0.77,0.98) | 1.52 (1.14,2.02) | 0.78 (0.68,0.89) | 0.82 (0.76,0.89) |
| 1-5 | 0.67 (0.64,0.70) | 0.98 (0.92,1.05) | 0.83 (0.80,0.87) | 0.71 (0.67,0.77) | 1.25 (1.06,1.47) | 0.73 (0.68,0.79) | 0.78 (0.75,0.82) |
| 5-25 | 0.72 (0.71,0.74) | 0.96 (0.93,0.99) | 0.88 (0.87,0.90) | 0.77 (0.75,0.79) | 0.94 (0.86,1.03) | 0.77 (0.74,0.80) | 0.81 (0.79,0.82) |
| 25-75 | 1.00 | 1.00 | 1.00 | 1.00 | 1.00 | 1.00 | 1.00 |
| 75-95 | 1.71 (1.69,1.72) | 1.15 (1.12,1.18) | 1.26 (1.25,1.28) | 1.42 (1.39,1.44) | 1.77 (1.66,1.89) | 1.74 (1.70,1.79) | 1.38 (1.36,1.40) |
| 95-99 | 3.54 (3.49,3.59) | 1.37 (1.32,1.43) | 1.76 (1.72,1.80) | 2.30 (2.25,2.35) | 5.51 (5.09,5.95) | 4.12 (4.00,4.24) | 2.28 (2.23,2.32) |
| >99 | 6.95 (6.83,7.08) | 1.62 (1.50,1.75) | 2.30 (2.21,2.39) | 3.29 (3.17,3.42) | 8.25 (7.32,9.31) | 10.04 (9.69,10.41) | 3.90 (3.77,4.02) |
| *Model 2: adjusted for demographic characteristics and morbidities* | | | | | | | |
| N | 3,156,863 | 3,123,591 | 3,070,812 | 3,153,179 | 3,153,179 | 3,155,971 | 3,105,540 |
| <1 | 0.79 (0.73,0.85) | 0.93 (0.81,1.06) | 0.92 (0.85,0.99) | 0.83 (0.74,0.94) | 1.47 (1.10,1.96) | 0.74 (0.64,0.85) | 0.83 (0.77,0.90) |
| 1-5 | 0.66 (0.63,0.69) | 1.02 (0.95,1.09) | 0.85 (0.82,0.89) | 0.73 (0.68,0.78) | 1.26 (1.07,1.48) | 0.72 (0.67,0.77) | 0.80 (0.77,0.83) |
| 5-25 | 0.73 (0.72,0.75) | 0.98 (0.95,1.02) | 0.90 (0.89,0.92) | 0.80 (0.78,0.82) | 0.97 (0.89,1.05) | 0.77 (0.74,0.80) | 0.82 (0.80,0.83) |
| 25-75 | 1.00 | 1.00 | 1.00 | 1.00 | 1.00 | 1.00 | 1.00 |
| 75-95 | 1.51 (1.49,1.52) | 1.07 (1.04,1.09) | 1.16 (1.15,1.18) | 1.26 (1.24,1.29) | 1.53 (1.43,1.64) | 1.55 (1.51,1.59) | 1.32 (1.30,1.34) |
| 95-99 | 2.29 (2.26,2.33) | 1.05 (1.01,1.10) | 1.34 (1.31,1.37) | 1.56 (1.52,1.60) | 3.41 (3.15,3.70) | 2.40 (2.33,2.48) | 1.98 (1.94,2.02) |
| >99 | 3.49 (3.42,3.55) | 1.16 (1.07,1.25) | 1.58 (1.52,1.65) | 1.93 (1.86,2.01) | 4.61 (4.06,5.23) | 3.80 (3.66,3.95) | 3.14 (3.04,3.25) |
| *Model 3: adjusted for demographic characteristics, morbidities and hemoglobin, WBC, and eGFR* | | | | | | | |
| N | 2,943,201 | 2,910,403 | 2,859,216 | 2,939,535 | 2,939,535 | 2,942,551 | 2,893,214 |
| <1 | 0.75 (0.69,0.81) | 0.96 (0.84,1.11) | 0.93 (0.86,1.01) | 0.80 (0.71,0.90) | 1.33 (1.00,1.77) | 0.68 (0.58,0.79) | 0.80 (0.74,0.87) |
| 1-5 | 0.65 (0.62,0.68) | 1.03 (0.96,1.10) | 0.86 (0.82,0.89) | 0.72 (0.67,0.77) | 1.50 (1.28,1.76) | 0.72 (0.66,0.78) | 0.80 (0.76,0.83) |
| 5-25 | 0.73 (0.72,0.75) | 0.99 (0.96,1.02) | 0.90 (0.89,0.92) | 0.80 (0.78,0.82) | 1.13 (1.03,1.23) | 0.79 (0.76,0.82) | 0.82 (0.81,0.84) |
| 25-75 | 1.00 | 1.00 | 1.00 | 1.00 | 1.00 | 1.00 | 1.00 |
| 75-95 | 1.47 (1.46,1.49) | 1.07 (1.04,1.10) | 1.16 (1.15,1.18) | 1.25 (1.23,1.27) | 1.05 (0.98,1.12) | 1.45 (1.41,1.49) | 1.30 (1.28,1.32) |
| 95-99 | 2.09 (2.06,2.12) | 1.08 (1.03,1.13) | 1.33 (1.30,1.36) | 1.45 (1.42,1.49) | 1.35 (1.25,1.47) | 1.97 (1.90,2.04) | 1.83 (1.80,1.87) |
| >99 | 2.81 (2.75,2.87) | 1.22 (1.12,1.32) | 1.56 (1.50,1.62) | 1.62 (1.56,1.69) | 1.38 (1.21,1.57) | 2.52 (2.41,2.64) | 2.61 (2.52,2.70) |
| *Sensitivity analysis – Model 4: adjusted for demographic characteristics, morbidities and hemoglobin, WBC, eGFR, albuminuria and serum albumin* | | | | | | | |
| N | 1,517,246 | 1,495,955 | 1,459,742 | 1,513,669 | 1,513,669 | 1,516,902 | 1,481,238 |
| <1 | 0.79 (0.72,0.87) | 1.01 (0.85,1.19) | 0.92 (0.83,1.01) | 0.76 (0.66,0.88) | 1.80 (1.35,2.40) | 0.62 (0.47,0.80) | 0.82 (0.75,0.89) |
| 1-5 | 0.62 (0.59,0.66) | 1.02 (0.93,1.11) | 0.87 (0.82,0.91) | 0.69 (0.64,0.75) | 1.38 (1.18,1.62) | 0.74 (0.65,0.85) | 0.80 (0.77,0.84) |
| 5-25 | 0.73 (0.72,0.75) | 0.99 (0.95,1.02) | 0.91 (0.89,0.93) | 0.79 (0.76,0.81) | 1.09 (1.00,1.19) | 0.80 (0.76,0.85) | 0.84 (0.82,0.86) |
| 25-75 | 1.00 | 1.00 | 1.00 | 1.00 | 1.00 | 1.00 | 1.00 |
| 75-95 | 1.40 (1.38,1.42) | 1.03 (1.00,1.06) | 1.14 (1.12,1.15) | 1.21 (1.19,1.24) | 0.97 (0.90,1.03) | 1.41 (1.36,1.47) | 1.24 (1.22,1.26) |
| 95-99 | 1.86 (1.83,1.89) | 1.04 (0.99,1.09) | 1.26 (1.23,1.29) | 1.36 (1.32,1.39) | 1.23 (1.14,1.34) | 1.77 (1.69,1.86) | 1.63 (1.59,1.67) |
| >99 | 2.34 (2.28,2.39) | 1.14 (1.04,1.25) | 1.47 (1.40,1.53) | 1.46 (1.39,1.52) | 1.07 (0.94,1.22) | 2.11 (1.99,2.23) | 2.16 (2.08,2.24) |
