# Supplementary material for: Red cell distribution width associations with clinical outcomes: A population-based cohort study
Source: PLoS One. 2019 Mar 13;14(3):e0212374. doi: 10.1371/journal.pone.0212374 (PMC6415845; doi:10.1371/journal.pone.0212374)
Supplement: S1 Data Availability Statement — (DOCX) [file pone.0212374.s005.docx]

Unfortunately, we are not able to make the data available to other researchers. We hold the data based on contractual agreements with Alberta Health, who is the data custodian. The custodian in turn is bound by its legal duties to not publicly share the data (i.e. to comply with Alberta law, and especially the Health Information Act). As specified by these agreements, any analyses of these data require ethics approval, as well as contracts for data use with Alberta Health.

While not the means by which we requested access to these data, possibly the most suitable portal for de novo data requests is found at <https://sporresources.researchalberta.ca>. Our request for data from Alberta Health predated the existence of this portal, so we instead met with the data custodians directly to negotiate a contract between our institution and the relevant arm of the provincial government, a process which took many months.

We do not know the specific criteria that would be applied to requests made through the above portal to legitimate researchers from within Alberta, or whether these data would be released to researchers outside of Alberta. The webpage provided also includes an email address where applicants can submit questions.

We provide below a list of the names for the variables that we used in this study, using the naming conventions that are used by Alberta Health:

Demographics (1 Apr 1994 to 31 Mar 2017) – pers_gender_code, pers_dob, death, death_date, out_migrate, out_migrate_date, reg_end_other, reg_end_other_date;

Registry (1 Apr 1994 to 31 Mar 2017) – year, pers_dob, addr_postal_code_fye, pers_reg_eff_date, pers_reap_end_date, ses_fye;

Provider Claims (1 Apr 1994 to 31 Mar 2017) – start_date, hlth_srvc_ccpx_code*, plcy_fee_mod_code_*, pers_capb_prvd_spec_ad;

Hospitalization Discharge Database (1 Apr 1994 to 31 Mar 2002) – start_date, end_date, sepi_dx_mr_icd9cm, sep_dx_oth_icd9cm_*, sepimrsp_dxtyp_hmri_*, serves_id_icd9_*, se_stdt_*;

Hospitalization Discharge Database (1 Apr 2002 to 31 Mar 2017) – start_date, end_date, dx*, type*, cci*, date*;

Ambulatory Care Classification System File (1 Apr 1997 to 31 Mar 2002) – start_date, sepi_dx_mr_icd9dm, sepi_dx_oth_icd9cm_*, serves_id_intrvntn_*, repi_fcentr_mis;

Ambulatory Care Classification System File (1 Apr 2002 to 31 Mar 2017) – start_date, icd*, cci*, sep_mis_acct_code;

Alberta Laboratory Values (1 May 2003 to 31 Mar 2017) – serum albumin, creatinine, hemoglobin, mean corpuscular volume, red cell distribution width, white blood count, urine dipstick, protein:creatinine ratio, albumin:creatinine ratio."
